# A survey of knowledge, attitude, and practices of private retail pharmacies staff in tuberculosis care: study from Dera Ismail Khan City, Pakistan

**DOI:** 10.1186/s40545-018-0134-1

**Published:** 2018-03-28

**Authors:** Tehmina Mustafa, Yasir Shahzad, Ayyaz Kiani

**Affiliations:** 1 Centre for International Health, Department of Global Public Health and Primary Care, University of Bergen, Postbox 7804, N-5020 Bergen, Norway; 2 Department of Thoracic Medicine, Haukeland University Hospital, Bergen, Norway; 3 Faculty of Pharmacy, Hamdard University, Islamabad, Pakistan

**Keywords:** Private retail pharmacies, Tuberculosis, DOTS, DI Khan City, Khyber Pakhtoon Khwa province, Pakistan

## Abstract

**Background:**

In order to engage pharmacies in tuberculosis (TB) care, a survey was conducted in the Dera Ismail (DI) Khan City of the Khyber Pakhtoon Khwa province, Pakistan. The objectives were to; 1) characterize the retail pharmacies; 2) determine knowledge of the staff on various aspects of pulmonary TB; 3) determine practices related to the sale of anti-TB drugs, and referrals of presumptive TB patient, and willingness to participate in the National Tuberculosis Control Programme’s (NTP) Directly Observed Treatment Short-Course (DOTS) strategy.

**Methods:**

A cross-sectional survey was conducted by using a structured questionnaire to collect data from pharmacy staff at all the private retail pharmacies of the DI khan city.

**Results:**

All the interviewed staff (*n* = 82) were males, only 38% had formal training as pharmacist (5%) or as a pharmacy assistant (33%). Pharmacies established for a longer period were better staffed and had high customer load. About 92% of the interviewed staff knew that persistent cough is a symptom for TB, 82% knew that TB is diagnosed by examination of sputum. Almost 66% of the pharmacy staff did not know multi-drug resistance TB as a consequence of improper treatment. Those with formal training and longer experience in retail pharmacy had better knowledge of various aspects of TB as compared to the staff with no formal pharmacy training and lesser experience (*p* < 0.01). Only 57% were aware of NTP while only 30% had heard of the DOTS strategy. All reported sale of first-line TB drugs as fixed dose combinations. The majority (80%) referred presumptive TB patients to chest physicians and no patient was referred to the NTP. Nearly 83% of the interviewed staff was willing to be involved in TB control efforts by getting training and referring patients to the DOTS facility.

**Conclusion:**

There was shortage of professionally qualified and female staff in private retail pharmacies. Knowledge of professionally qualified staff about TB seemed sufficient to identify presumptive TB patients; however, their knowledge about NTP and DOTS was poor, and referral practices to NTP and DOTS centers were suboptimal. Majority of staff was willing to be involved in TB control efforts.

## Background

Pakistan is among the top six high tuberculosis (TB) burden countries in the world. The National Tuberculosis Control Programme (NTP) of Pakistan has implemented the World Health Organisation (WHO) endorsed Directly Observed Treatment Short-Course (DOTS) strategy for TB control, and in 2005 attained near universal DOTS coverage in all the districts within the public sector [1]. Despite country-wide DOTS coverage the incidence of TB remains high [2, 3]. It is mostly acknowledged that private sector provides health care to about 70% of the population, while public health sector caters only 30% of the population [4]. The NTP of Pakistan has made significant progress in involving diverse private health care providers in TB care and control through private-public partnership programme resulting in an increase in case detection and treatment [4–8].

Retail pharmacy staff and pharmacists are an important part of the private health-care sector in Pakistan which has not been engaged in the TB care, prevention and control. Retail pharmacies usually have close links to the society, and not only dispense prescriptions for anti-TB drugs, but are often the first point of contact for people with symptoms of TB. Experiences in many countries have revealed that with proper training, the retail pharmacy staff has the potential to play an important role in TB case management and detection [9]. WHO has recognized pharmacists as one of the six pillars for providing efficient TB care with International Pharmaceutical Federation and have stressed the need for intense collaborative exercise [10].

Some efforts have been made towards engaging the pharmacies in TB care in Pakistan in the Karachi city located in the Southern province [11]. The Khyber Pakhtoon Khwa province located in the north of Pakistan is considered a hot spot of TB due to large refugee population. The real TB cases are estimated to be higher than the notified TB cases as suggested by a recent prevalence survey conducted in Pakistan [3]. In order to improve TB care by involving pharmacies, we conducted a survey in the DI Khan City of the province to; 1) characterize the retail pharmacies; 2) determine the knowledge of the retail pharmacy staff on various aspects of TB disease, treatment, NTP and the DOTS; 3) determine practices related to the sale of anti-TB drugs, identification and referrals of presumptive TB patient, and willingness of the pharmacy staff to participate in the NTP’s DOTS strategy.

## Methods

### Study design and setting

The study was a cross-sectional survey conducted in the DI Khan city, Pakistan, during October–December 2014. DI Khan is one of the south most district of Khyber Pukhtoon Khwa province with a population of about 1.2 million. The annual TB notification rate for NTP in this province was 84/100,000 and TB prevalence 177/100,000 in 2011 [3]. List of private retail pharmacies in the area was obtained and all 93 pharmacies were intended to be included in the study. Homeopathic pharmacies were excluded. Data was collected by interview of the pharmacy staff using a structured questionnaire. The pharmacy staff included pharmacists holding a 5 year professional degree, pharmacy assistants with a 2 years formal training, and sales persons who had not received any formal training in pharmacy. The drugs were dispensed by any of the above mentioned persons. The retail outlets have generally high staff turnover and therefore one staff member with the longest experience from each pharmacy was interviewed. Questionnaire was adopted from an earlier study [12] and had 3 parts; 1) General information & retail pharmacy profile − 17 questions, 2) Knowledge about tuberculosis and NTP-DOTS strategy − 15 questions, 3) Action and practices in TB case detection- 19 questions. Questionnaire was assessed for its content by the authors based on their experience in tuberculosis and pharmacy in consultation with their colleagues expert in the field. The questionnaire was also validated for clarity and ease of understanding by pre-testing it with four respondents before data collection.

### Ethical considerations

The protocol was exempted from the requirement of ethical clearance from the Regional Ethical Committee of Norway, and was approved by the National Bioethical Committee of Pakistan, (Ref: No. 4–87/14/NBC-156/RDC/477). At each pharmacy permission was taken from the owner of the pharmacy and the main seller (if they were different) by administering the information sheet containing the necessary information about the study, voluntary participation, confidentiality and anonymity. Further elaboration was done by the interviewer if required. Participants were asked to sign the consent form.

### Data management and analysis

Data was entered, checked for accuracy, cleaned and analysed in Statistical Package for the Social Sciences (SPSS) version 22. Descriptive statistics were used. Fischer’s exact test was used to determine the significance of association between categorical variables. Pearson correlation coefficient was used to measure the linear correlation between continuous variables. The level of significance was set at 5%.

## Results

Of the 93 pharmacies, 82 were included. The owners of 7 pharmacies did not agree to be interviewed while 4 pharmacies were included in pre-test and not included in the results.

### Characteristics of the pharmacies

**Fig. 1:**
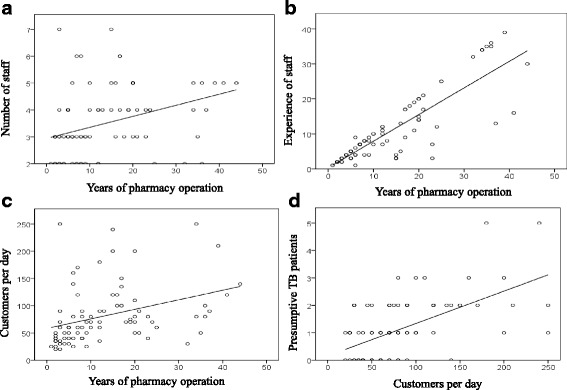
Characteristics of pharmacies with longer duration of operation. Pharmacies with a longer duration of operation had higher staff number (*p* = 0.002) (**a**), higher number of more experienced staff in retail pharmacy (*p* = 0.000) (**b**), and the higher number of customers per day (*p* = 0.001) (**c**). The pharmacies with higher customer load reported to have higher number of presumptive TB patients (*p* = 0.000) (**d**). Significance was determined by using the Pearson’s correlation test

The characteristics of pharmacies are summarized in Table 1. All pharmacy staff was male, median age was 33.5 years (range19–64 years). Seventy six percent of the pharmacies were quite close to the health facility within a walking distance of 10 min, and TB DOTS center were also located in close proximity of health facilities. The duration of operation of the pharmacies had a direct positive correlation with the number of staff (*p* = 0.002), their experience in retail pharmacy (*p* = 0.000), and customer load (*p* = 0.001) as shown in Fig. 1a-b. The frequency of reported presumptive TB patients varied among pharmacies. Pharmacies with longer duration of operation had a higher customer load and those with higher customer load reported higher number of presumptive TB patients as shown in Fig. 1c-d.

**Table 1:** Characteristics of the private retail pharmacies in the DI Khan city, Pakistan

	Number (%)
Total Participants	82 (100)
Professional background	
Pharmacists	4 (5)
Pharmacy assistants	27 (33)
Sales person	51 (62)
Experience of staff in retail pharmacy (years)	
≤ 5	34 (41)
6–15	30 (37)
16–25	10 (12)
> 25	8 (10)
Number of staff working in pharmacies	
2–3	47 (57)
4–5	29 (35)
6–7	6 (7)
Walking distance of health facility from pharmacy	
< 10 min	62 (76)
< 30 min	20 (24)
Years of pharmacies operation	
0–5	24 (29)
6–15	29 (35)
16–25	18 (22)
> 25	11 (13)
Number of customers per day	
≤ 50	31 (38)
51–100	33 (40)
> 100	18 (22)
Records for customers	
Sales/drug register	11 (13)
No record	71 (87)
Availability of TB informational materials	
Brochures/pamphlet	10 (12)
Posters	11 (13)
Pharma companies drug advertisement	10 (12)
No Information material	51 (62)

### Knowledge of pharmacy staff about TB and NTP

The majority knew that TB spreads through presence of untreated TB patients in community (93%), and poverty (72%) but limited number knew that TB spreads through poor ventilation (50%) and overcrowding (28%). Majority (92%) identified persistent cough as TB symptom, while other symptoms were less frequently (25–61%) identified. Majority knew that TB can be prevented by curing TB patients (93%), and by covering mouth and nose of TB patient during coughing or sneezing (78%). The majority (82%) knew that TB is diagnosed by examination of sputum. Almost 66% did not know about multidrug resistant (MDR)-TB and factors leading to MDR-TB. Only 57% were aware of NTP while only 30% had heard of the TB-DOTS.

Table 2 summarizes the knowledge of pharmacy staff on various aspects of TB and shows relationship with the professional background and retail pharmacy experience. The respondents were grouped as knowing and not knowing if they could answer the key questions presented in the Table. A significant positive association was observed between formal training in pharmacy and knowledge of TB on several aspects. Pharmacists had better knowledge about TB as compared to pharmacy assistants and sales persons, and pharmacy assistants had better knowledge as compared to sales persons. A significant positive association was also observed between several aspects of knowledge of TB and the duration of work experience.

**Table 2:** Knowledge of the retail pharmacy staff about tuberculosis (TB) and correlation with the professional background and duration of working experience in the DI Khan city, Pakistan. Statistical significance is determined by using Fisher’s Exact test

Professional background & Experience in years	Know^a^ n (%^a^)	Do not know^a^ n (%)	*P*-value
	Exposure: Breathing air containing TB causing microorganisms		
Pharmacists	4 (100)	0 (0)	0.071
Pharmacy assistants	25 (93)	2 (7)	
Sales person	37 (73)	14 (27)	
≤ 5 years	19 (56)	15 (44)	0.000
6–15 years	29 (97)	1 (3)	
> 15 years	18 (100)	0 (0)	
	Transmission: Poor ventilation; overcrowding; presence of untreated TB patients in the house/community		
Pharmacists	4 (100)	0 (0)	0.451
Pharmacy assistants	22 (82)	5 (19)	
Sales person	36 (71)	15 (29)	
≤ 5 years	20 (59)	14 (41)	0.008
6–15 years	25 (83)	5 (17)	
> 15 years	17 (94)	1 (6)	
	Symptoms: Persistent cough; fever; sweat		
Pharmacists	4 (100)	0 (0)	0.000
Pharmacy assistants	15 (57)	12 (44)	
Sales person	9 (18)	42 (82)	
≤ 5 years	5 (15)	29 (85)	0.001
6–15 years	11 (37)	19 (63)	
> 15 years	12 (67)	6 (33)	
	Prevention: Covering mouth and nose when patient cough or sneeze; by curing TB patients		
Pharmacists	4 (100)	0 (0)	0.213
Pharmacy assistants	21 (78)	6 (22)	
Sales person	32 (63)	19 (37)	
≤ 5 years	16 (47)	18 (53)	0.000
6–15 years	23 (77)	7 (23)	
> 15 years	18 (100)	0 (0)	
	Diagnosis: Sputum smear microscopy		
Pharmacists	4 (100)	0 (0)	0.103
Pharmacy assistants	25 (93)	2 (7)	
Sales person	38 (75)	13 (26)	
≤ 5 years	24 (76)	10 (29)	0.032
6–15 years	25 (83)	5 (17)	
> 15 years	18 (100)	0 (0)	
	Consequences of improper treatment: Drug resistance, Disease deteriorates, Disease spread to others, Relapse^b^		
Pharmacists	4 (100)	0 (0)	0.002
Pharmacy assistants	12 (44)	15 (56)	
Sales person	11 (22)	40 (78)	
≤ 5 years	5 (15)	29 (85)	0.001
6–15 years	10 (33)	20 (67)	
> 15 years	12 (68)	6 (33)	
	MDR-TB: Improper treatment, failure to complete treatment, Presence of patients with MDR-TB in household/community^c^		
Pharmacists	4 (100)	0 (0)	0.003
Pharmacy assistants	9 (33)	18 (67)	
Sales person	10 (20)	41 (80)	
≤ 5 years	7 (21)	27 (79)	0.360
6–15 years	9 (30)	21 (70)	
> 15 years	7 (39)	11 (61)	
	Duration of TB treatment: 6–9 months		
Pharmacists	4 (100)	0 (0)	1.00
Pharmacy assistants	26 (96)	1 (4)	
Sales person	48 (94)	3 (6)	
≤ 5 years	31 (91)	3 (9)	0.535
6–15 years	29 (97)	1 (3)	
> 15 years	18 (100)	0 (0)	

Decimal value ≥0.5 is rounded up to 1.0. ^a^ The respondents were grouped as knowing and not knowing if they could answer the questions mentioned by key words in table. ^b^ 3/4, ^c^ 2/3 answers were grouped as knowing. MDR-TB: Multi-drug resistant tuberculosis

### Attitude and practices related to referral of the presumptive TB patients and sale of anti-TB drugs (Table 3)

The majority of the staff (80%) referred the presumptive TB patients to chest physicians, and none referred patients to the NTP’s DOTS centres. None of the staff had received any training relevant to TB in last 2 years. Majority (66%) of staff had a positive attitude towards getting more information about NTP and 83% of pharmacy staff was willing to be involved in TB control efforts by getting training and referring patients to the DOTS facility. All the staff informed that anti-TB drugs were dispensed according to the prescription and none of the staff reported dispensing anti-TB drugs without prescription. All prescribed drugs were reported to be a combination preparation of 4 drugs (4-FDC-RHZE) or 2 drugs (2-FDC-RH) with correct duration of treatment. However, due to low purchasing capacity of patients, drugs were usually (52%) dispensed for shorter duration.

**Table 3:** Practices of the retail pharmacy staff related to identification and referral of the presumptive tuberculosis (TB) patients and sale of anti-TB drugs in the DI Khan city, Pakistan

	Number (%)
Total participants	82 (100)
Suspected TB patients per week	
0	28 (34)
1–2	47 (57)
3–5	7 (9)
Referral of suspected TB patients	
Nearby laboratory	22 (27)
Nearby GP	19 (23)
Chest physician	65 (79)
TB DOTS centre	0 (0)
No referral	
Dispense broad spectrum antibiotics	11 (13)
Dispense cough syrup	28 (34)
Dispense anti-TB drugs	1 (1)
TB patients with a doctor’s prescription	82 (100)
Prescriped duration of anti-TB drugs	
6 months	62 (76)
9 months	16 (19)
Do not know	4 (5)
Number of prescribed anti-TB drugs	
2 medicines	25 (30)
3 medicines	39 (48)
4 medicines	18 (22)
Vitamin B6 supplement in prescription	73 (89)
Frequently sold TB medicines	
4-FDC-RHZE combination	82 (100)
3-FDC-RHE combination	7 (8)
2-FDC-RH combination	79 (96)
Isoniazid	1 (1)
Pyrazinamide	0 (0)
Ethambutol	0 (0)
Streptomycin	28 (34)
Kanamycin	4 (5)
Patients buy anti-TB medicines	
1 month	7 (8)
2 months	11 (13)
3 months	22 (27)
4 months	3 (4)
6 months	35 (43)
9 months	4 (5)

## Discussion

Findings of our study show that the pharmacy staff encounters presumptive TB patients on daily basis and despite their low buying capacity they were referred to the specialized chest physicians, requiring payment, instead of TB-DOTS centers where they could get free-of-cost TB care. The most plausible explanation of this practice seems to be the insufficient knowledge about the TB care provided by the NTP’s DOTS strategy. Other possible explanations could be lack of trust on quality of medicine and easy access to private sector. However this needs further investigation as pharmacies have a potential to improve the situation by imparting right information to the public. The majority of the retail pharmacy staff wanted to be involved in the NTP’s TB care efforts. However proper training is a necessary pre-requisite as our survey revealed that none of the pharmacy staff had received any training relevant to TB during the last 2 years, and informational material relevant to TB were present in very limited numbers of pharmacies. Pharmacy staff was willing to receive relevant training for this purpose. A study from India has shown that retail pharmacy staff can contribute towards imparting TB knowledge and care by getting training through collaborative NTP or professional association workshops [13].

An important finding of our study was shortage of qualified personnel in private retail pharmacies. Patients were mainly handled by unqualified salesmen who may give unsatisfactory consultation. Similar findings have also been reported by some earlier studies carried out in other cities of Pakistan [14–16]. Thus, there is a need for implementation of law to guarantee presence of qualified personnel in private retail pharmacies which would result in better patient care oriented services.

Our study showed that pharmacies which were established for a longer period were better staffed and had high customer load, implying better contact with- and trust of public, so that people use them more often as consultation sites as compared to newly established pharmacies. Such pharmacies could serve as sites for TB-DOTS consultation and care.

There was no female retail pharmacy staff in our study area. This can be related to social and cultural factors in this region of Pakistan where gender roles define that females do not generally participate in sales and marketing professions. This could have some implications in interaction of the staff with the female presumptive TB patients and it would be an advantage to have some balance in the gender distribution of the pharmacy staff.

This research study did not permit evaluation of the frequency of sales of anti-TB drugs without prescription, and therefore we could not validate the finding that anti-TB drugs are not sold without a prescription. The majority of anti-TB drugs present in the pharmacies were fixed dose combinations of either four or two first line anti-TB drugs thereby reducing the probability of over-the-counter delivery of anti-TB drugs as broad spectrum antibiotics for other purposes.

A weakness of the study is selection of only one experienced respondent from each pharmacy. This could have added bias to the study. The retail outlets have generally high staff turnover and therefore it was reasonable to interview such staff who have a longevity and most likely to deal with targeted cases. The respondents varied in experience across the pharmacies reducing the biasness due to length of experience to some extent.

## Conclusion

This study shows that there was shortage of professionally qualified and female staff in the private retail pharmacies. Knowledge of professionally qualified staff about TB seemed sufficient to identify presumptive TB patients; however, their knowledge about NTP and DOTS was poor, and referral practices to NTP and DOTS centers were suboptimal. Majority of staff was willing to be involved in TB control efforts and receive training, thereby representing a significant resource for improving TB care.

## Abbreviations

DOTS: Directly Observed Treatment Short Course
NTP: National Tuberculosis Control Programme
TB: Tuberculosis
WHO: World Health Organisation
